# CD9 Tetraspanin: A New Pathway for the Regulation of Inflammation?

**DOI:** 10.3389/fimmu.2018.02316

**Published:** 2018-10-09

**Authors:** Carole Brosseau, Luc Colas, Antoine Magnan, Sophie Brouard

**Affiliations:** ^1^Centre de Recherche en Transplantation et Immunologie UMR 1064, INSERM, Université de Nantes, Nantes, France; ^2^Institut de Transplantation Urologie Néphrologie, CHU Nantes, Nantes, France; ^3^Institut du Thorax, Plateforme Transversale d'Allergologie, CHU de Nantes, Nantes, France; ^4^Institut du thorax, Inserm UMR 1087, CNRS UMR 6291, Université de Nantes, Nantes, France

**Keywords:** CD9, tetraspanin, inflammation, hematopoietic cells, cancer, lung allograft dysfunction, infections, hypersensitivity

## Abstract

CD9 belongs to the tetraspanin superfamily. Depending on the cell type and associated molecules, CD9 has a wide variety of biological activities such as cell adhesion, motility, metastasis, growth, signal transduction, differentiation, and sperm–egg fusion. This review focuses on CD9 expression by hematopoietic cells and its role in modulating cellular processes involved in the regulation of inflammation. CD9 is functionally very important in many diseases and is involved either in the regulation or in the mediation of the disease. The role of CD9 in various diseases, such as viral and bacterial infections, cancer and chronic lung allograft dysfunction, is discussed. This review focuses also on its interest as a biomarker in diseases. Indeed CD9 is primarily known as a specific exosome marker however, its expression is now recognized as an anti-inflammatory marker of monocytes and macrophages. It was also described as a marker of murine IL-10-competent Breg cells and IL-10-secreting CD9^+^ B cells were associated with better allograft outcome in lung transplant patients, and identified as a new predictive biomarker of long-term survival. In the field of cancer, CD9 was both identified as a favorable prognostic marker or as a predictor of metastatic potential depending on cancer types. Finally, this review discusses strategies to target CD9 as a therapeutic tool. Because CD9 can have opposite effects depending on the situation, the environment and the pathology, modulating CD9 expression or blocking its effects seem to be a new promising therapeutic strategy.

## CD9 is a tetraspanin family member

Tetraspanin proteins are ubiquitously expressed and consist of 4 transmembrane domains, an intracellular terminus and 2 extracellular loops (1). Tetraspanins function as organizers of the cell surface by recruiting specific partner proteins into tetraspanin-enriched microdomains (TEMs) (2). Bringing together a large variety of molecules and amplifying their activities, tetraspanins regulate various cellular processes, such as cell adhesion, motility, growth, differentiation, signal transduction, and sperm–egg fusion (3–5) (summarized Table 1).

**Table 1:** Functions of CD9 on different cell populations.

	**Functions of CD9**	**References**
	Cell migration and invasion	Hadjiargyrou et al. (6); Powner et al. (7)
	Mice fertility	Le Naour et al. (8)
	Sperm-egg fusion	Le Naour et al. (8)
Hematopoietic stem cell and progenitor cell	Megakaryocytic differentiation	Clay et al. (9)
	Production of myeloid cells	Oritani et al. (10)
	Adhesion between myeloid and stromal cells	Aoyama et al. (11)
Myeloid lineages	Chemotaxis of mast cells	Qi et al. (12)
	Non-immunoglobulin E (IgE)-mediated mast cell activation	Redegeld et al. (13)
	Degranulation and Ca2+ release of mast cells	Hálová et al. (14)
	Basophils degranulation	Higginbottom et al. (15)
	Eosinophils and platelets degranulation	Fernvik et al. (16)
	CD4^+^ T-cell activation, proliferation and cytokine production by eosinophils	Kim et al. (17); Akuthota et al. (18)
	Release of IL-12 by eosinophils	Bandeira-Melo et al. (19)
	Platelet aggregation and granule release	Qi et al. (20)
	Negative regulator of lipopolysaccharide-induced macrophage activation	Suzuki et al. (21)
	Macrophages activation	Kaji et al. (22)
	Secretion of cytokines by macrophages	Ha et al. (23)
	Foam macrophages formation	Huang et al. (24)
Dendritic cells	IL-10 production	Zilber et al. (25)
	HLA-DR signaling activation	Zilber et al. (25)
	Regulation of MHC-II intracellular trafficking	Rocha-Perugini et al. (26)
	Dexosomes formation	Rocha-Perugini et al. (26)
B cells	Tyrosine phosphorylation of different proteins via CD19 association	Horváth et al. (27)
	B cell differentiation by modulating integrin activity	Shaw et al. (28)
	Adhesion of B cells to follicular dendritic cells	Yoon et al. (29)
	Survival of human germinal center B cells	Yoon et al. (29)
	May control antibody production	van Spriel et al. (30)
	Enhancement and maintenance of IL-10 secretion	Ha et al. (23); Kabuto et al. (31)
T cells	Proliferation of virgin T cells	Tai et al. (32)
	Induction of apoptosis of once-activated T cells	Tai et al. (32)
	T cells differenciation into type 2 effector cells	Serra et al. (33)
	Self-antigen- and recall antigen-induced T cell activation	Kobayashi et al. (34)
	Integrin-mediated signaling	Rocha-Perugini et al. (26)
	Membrane-phosphatidylserine exposure	Li et al. (35)
Endothelial cells	Endothelial–leucocyte adhesion	Nourshargh et al. (36)
	Extravasation	Bailey et al. (37)

CD9 (also known as TSPAN29, Leukemia-Associated Cell Surface Antigen p24, and Motility-Related Protein-1) is a member of the tetraspanin superfamily. CD9 was first identified by Kersey et al. as the human lymphohematopoietic progenitor cell surface antigen p24 using a monoclonal antibody that bound to acute lymphoblastic leukemia cells (38). Characterization of CD9 revealed its expression on a large variety of hematopoietic and non-hematopoietic cells, such as stromal cells, megakaryocytes, platelets, B and T lymphocytes, dendritic cells, endothelial cells, mast cells, eosinophils, and basophils (16, 27, 39). Tetraspanin CD9 was first described as a motility-related factor (40) and subsequently was associated with various integrin adhesion receptors regulating cell migration and invasion (6, 7). Homozygous adult CD9^−/−^ mice exhibit no obvious abnormalities. However, when the CD9^−/−^ mice are intercrossed, fertility is severely reduced (8). CD9 thus appears to be essential for sperm-egg binding or fusion but the exact mechanism is still not fully understood. CD9 is localized to the microvillar region of the oocyte and may be necessary in organizing multiprotein complexes. Some studies highlighted CD9 interactions with other membrane proteins necessary for fertilization such as pregnancy specific glycoprotein 17 (PSG17) (41) (waterhouse jexpmed 2002) or beta1 integrins (42) (chen pnas 1999). Interaction of CD9 with the integrin alpha6beta1 seems to be essential for strong IZUMO1-dependent adhesion of sperm with the oocyte (43) (inoue develipment 2013). CD9 may play also a role in generating fusion competent sites on the egg membrane required for the fusion and in sperm-adhesion strengthening (44) (jégou pnas)

Regarding pathological models, CD9-knockdown suppresses the invasive and metastatic capacity of breast cancer cells in mouse xenografts (45), and CD9 deficiency reduces the severity of osteoarthritis in aging and antigen-induced arthritis models (46); however, CD9 deficiency also induces a phenotype similar to human chronic obstructive pulmonary disease (COPD) (47). These reports clearly suggest that CD9 is crucial in the control of inflammation and support its role in the modulation of humoral immune responses (30), the production of myeloid cells (10) and the secretion of cytokines by macrophages (23).

## CD9 is involved in hematopoietic stem cell and progenitor cell differentiation

**Figure 1:**
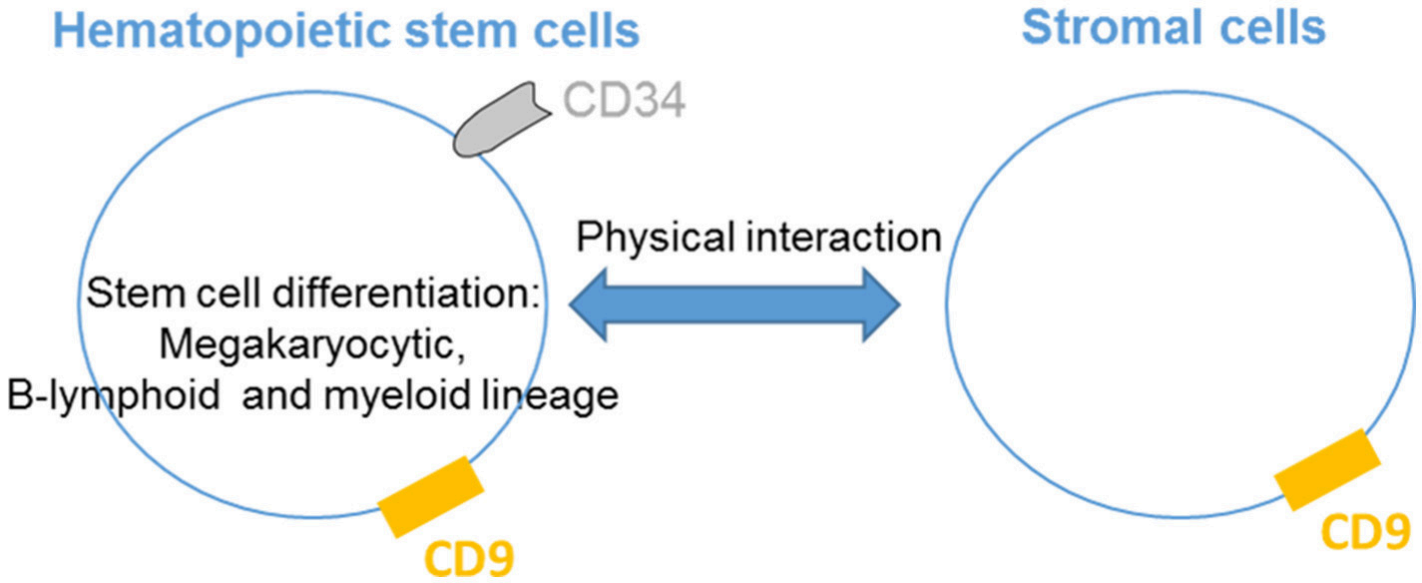
CD9 regulates hematopoietic stem cells differentiation. CD9 is expressed by hematopoietic stem cells and is involved in the differentiation of the megakaryocytic, B-lymphoid and myeloid lineages. CD9 expressed in stromal cells influences physical interactions with hematopoietic cells.

CD9 is present on hematopoietic cells as well as cells that regulate their activities in the bone marrow and critical hematopoiesis events (Figure 1). Thus, CD9 is expressed in nearly all CD34^+^ bone marrow cells with a range of expression that varies from low to high levels according to the degree of cell differentiation (9). CD34^+^CD10^+^CD19^+^ early B cells express high levels of CD9 correlating with the presence of the early megakaryocytic marker CD41/GPIIb, indicating that cells with the highest levels of CD9 are committed to the B-lymphoid and megakaryocytic lineages. The early increase in CD9 expression during megakaryopoiesis suggests that CD9 may play a role in megakaryocytic differentiation by participating in the membrane remodeling process. Indeed, antibody ligation of CD9 alters the *in vitro* differentiation of human CD34^+^ cells into megakaryocytes. The production of myeloid cells in long-term bone marrow cultures is blocked by the addition of anti-CD9 KMC8.8 (10), and the ligation of CD9 promotes adhesion between myeloid and stromal cells. Finally, pluripotent hematopoietic cells cultured with stromal cells in the presence of anti-CD9 KMC8.8 migrate beneath the adherent stromal cell layer and have undifferentiated properties (11). Altogether, these data demonstrate that stromal cells expressing CD9 influence physical interactions with hematopoietic cells and may be one factor that determines the degree of stem cell differentiation.

## CD9 is involved in the regulation of the myeloid lineages

CD9 is abundantly expressed on the plasma membrane of different myeloid lineage cells such as mast cells (48), basophils (15), eosinophils (16), and macrophages (24).CD9 has a role in the cytokine-mediated chemotactic response of human mast cells. Chemotaxis of mast cells toward interleukin-16 (IL-16) is abrogated by anti-CD9 antibodies and decreased expression of CD9 using RNA interference; these results demonstrate that CD9 acts as an substitute IL-16 receptor (12). Moreover, CD9 induces non-immunoglobulin E (IgE)-mediated mast cell activation (13). In mast cells, CD9 co-localizes with the high-affinity IgE receptor FcεRI and non-T-cell activation linker (NTAL). Antibody-mediated cross-linking of CD9 activates mast cells, causing degranulation, calcium release and tyrosine phosphorylation of various proteins, such as NTAL (14). Thus, CD9 activates mast cells in a different way from the stem cell factor and IgE mediation.

CD9 is also expressed on basophils, and in the same manner as mast cells, antibody cross-linking of CD9 and FcεRI stimulates degranulation. In a model of rat basophilic leukemia cells, transfected human CD9 cells degranulate in response to anti-CD9 antibodies co-ligated with FcεRI (15). Expression of CD9 is a feature of both eosinophils and platelets, and antibody cross-linking of CD9 activates the degranulation of eosinophils and platelets through integrins and FccRIIa, respectively (16). Interestingly, this cross-linking induces eosinophil degranulation and enhances survival. Localization of CD9 with MHC Class II on eosinophil plasma membrane is necessary for the ability of eosinophils to trigger CD4^+^ T-cell activation, proliferation and cytokine production (17, 18). Finally, stimulation of eosinophils through CD9 triggers the release of IL-12 by a process of vesicular transport, suggesting a possible function for CD9 in tempering the Th2 cell-dependent inflammatory response (19). Interestingly, CD9 antibodies induce platelet aggregation and granule release, which is dependent on FccRIIa, although the signal generated is distinct from FccRIIa activation alone (20). In contrast, neutrophil degranulation is not provoked by the blockade of CD9, consistent with a lack of expression of CD9 on neutrophils (17).

CD9 tetraspanin is expressed differentially by monocyte subsets, with higher levels on CD14^++^CD16^−^ subsets than on CD14^++^CD16^+^ and CD14^+^CD16^++^ monocytes (49). Maturation of monocytes results in increased CD9 expression with even higher levels present in monocyte-derived macrophages. Furthermore, CD9 expression on monocyte-derived macrophages is stimulated by M-CSF and decreased by interferon-γ or HIV-1 infection (50). However, Suzuki et al. describe CD9 as a negative regulator of lipopolysaccharide-induced macrophage activation and lung inflammation because deletion of CD9 in mice enhances macrophage infiltration and TNF-α production in the lung after intranasal administration of LPS *in vivo* (21). Consistent with the immune cells described above, the anti-CD9 antibody activates macrophages. KMC8.8 treatment of mouse macrophages induces protein tyrosine phosphorylation, filopodium extension, and cell aggregation caused by FcγRIIB/III-CD9 co-cross-linking (22); CD9 functionally associates with FcγRs and modifies signals for phagocytosis and inflammatory responses. Finally, CD9 is associated with CD36 on the surface of macrophages, leading to foam cell formation in response to oxidized low-density lipoproteins (24).

**Figure 2:**
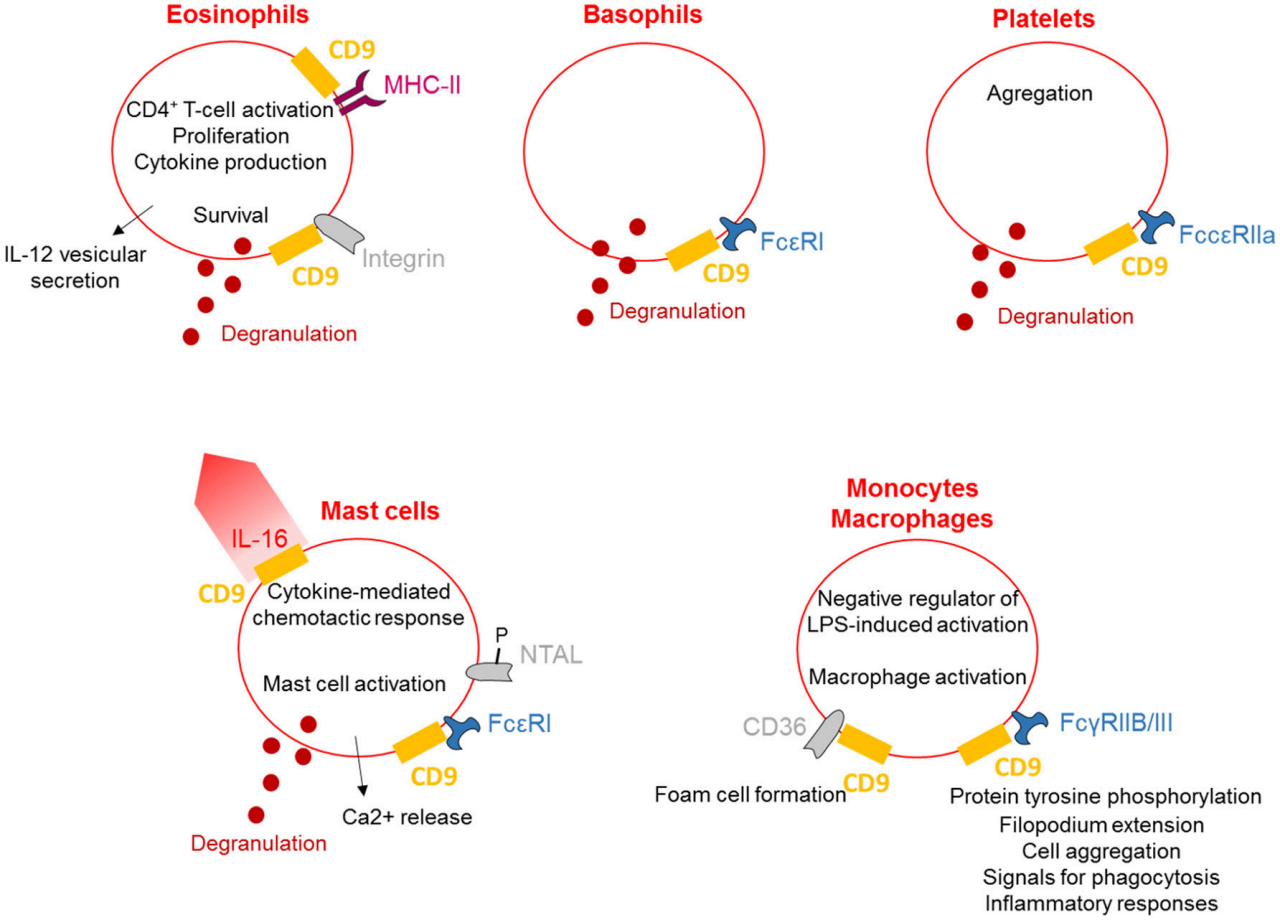
CD9 regulates myeloid lineages. CD9 is expressed on the plasma membrane of different myeloid lineages and is associated with MHC-II and integrins on eosinophils and with FcRs on basophils, platelets, mast cells and macrophages. CD9 activates cells degranulation and cytokines secretion and is involved in cytokine-mediated chemotactic responses. Finally, CD9 regulates inflammation, allowing eosinophils to stimulate CD4^+^ T-cell activation and induce macrophage activation.

Together, these data demonstrate that crosslinking of CD9 induces mast cell, basophil, eosinophil and platelet activation, leading to their degranulation, and also stimulates a range of activities such as chemotaxis and cytokine release (Figure 2). Activation of macrophages can also occur via CD9 crosslinking to allow the regulation of inflammation.

## CD9 is essential for dendritic cells to regulate inflammation

**Figure 3:**
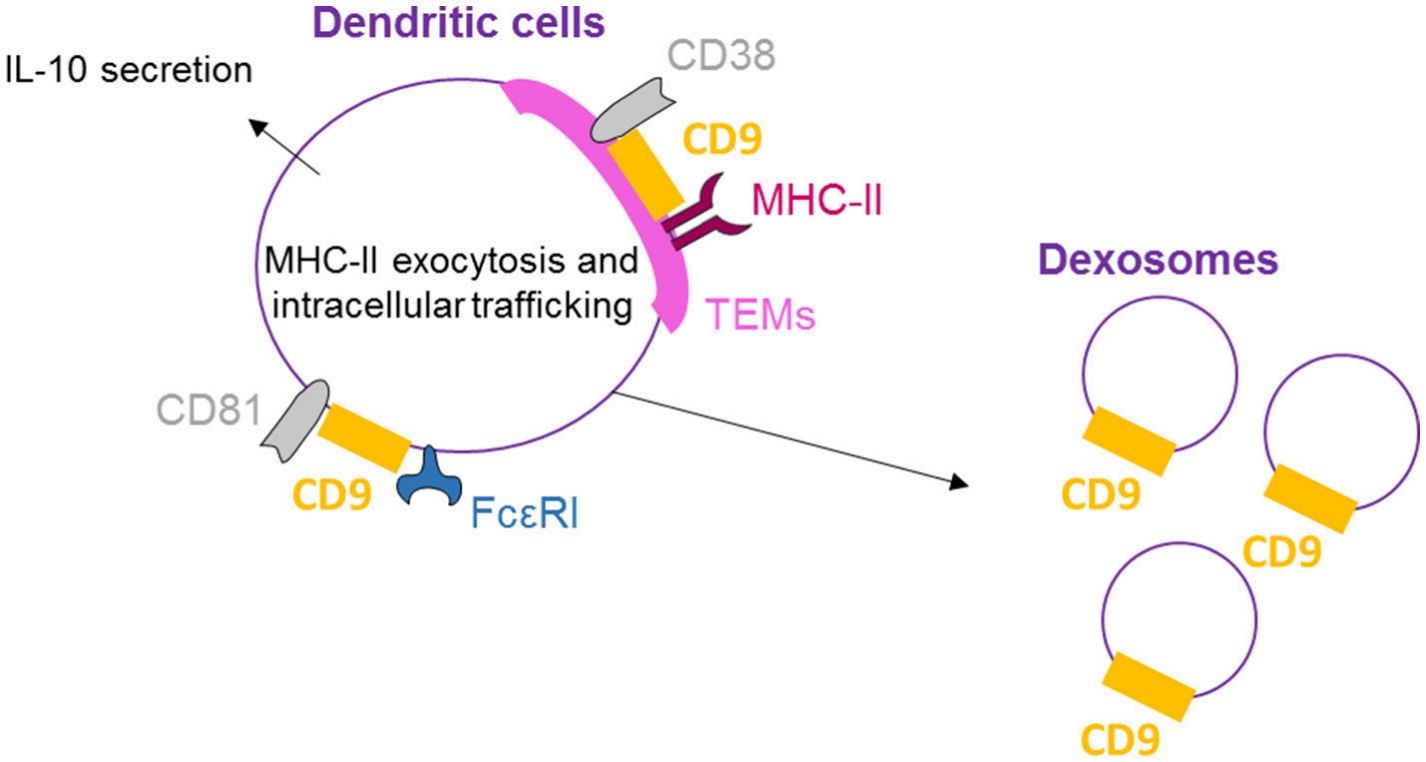
CD9 is necessary for dendritic cell activity. On human DCs, CD9 co-localizes with CD81 and FcεRI, and crosslinking with FcεRI induces IL-10 secretion. CD9 is also physically associated with MHC-II molecules and CD38 in TEMs, and the integrity of these lipid rafts is necessary for antigenic presentation by DCs and dexosome release. CD9 is now mostly known as a specific exosome marker.

CD9, CD81, and FcεRI are co-expressed and co-localized in human dendritic cells (DCs) as shown by Peng et al. by flow cytometry, confocal microscopy, and immunoprecipitation/immunoblotting experiments (51) (Figure 3). Concomitant activation by FcεRI and CD9 crosslinking results in increased IL-10 production, highlighting the functional cooperation between CD9 and FcεRI. Also in DCs, MHC-II molecules, CD38 and CD9 are physically associated in TEMs (highlighted by co-immunoprecipitation and co-capping experiments), and the integrity of these lipid rafts is necessary for HLA-DR signaling activation (25). With respect to DC function, CD9 knockout in DCs induces reduced levels of T-cell activation than wild-type DCs (26). This effect is related to a reduction in MHC-II surface expression in CD9-deficient DCs due to an impairment of exocytosis. Internalization is also blocked, demonstrating that CD9 specifically regulates antigenic presentation in DCs through the regulation of MHC-II intracellular trafficking. Dendritic cells release large quantities of exosomes, known as dexosomes, to amplify both the adaptive (T-cells) and innate (natural killer cells) cellular immune responses; these dexosomes comprise a characteristic set of proteins, including CD9, all the known antigen-presenting molecules (MHC-I and II, CD1 a, b, c, and d) and the costimulatory molecule CD86 (52). CD9 crosslinking thus activates DCs, leading to cytokine production, and is necessary for antigenic presentation essential for the regulation of inflammation by DCs.

## CD9 is involved in the regulation of the lymphoid lineages

**Figure 4:**
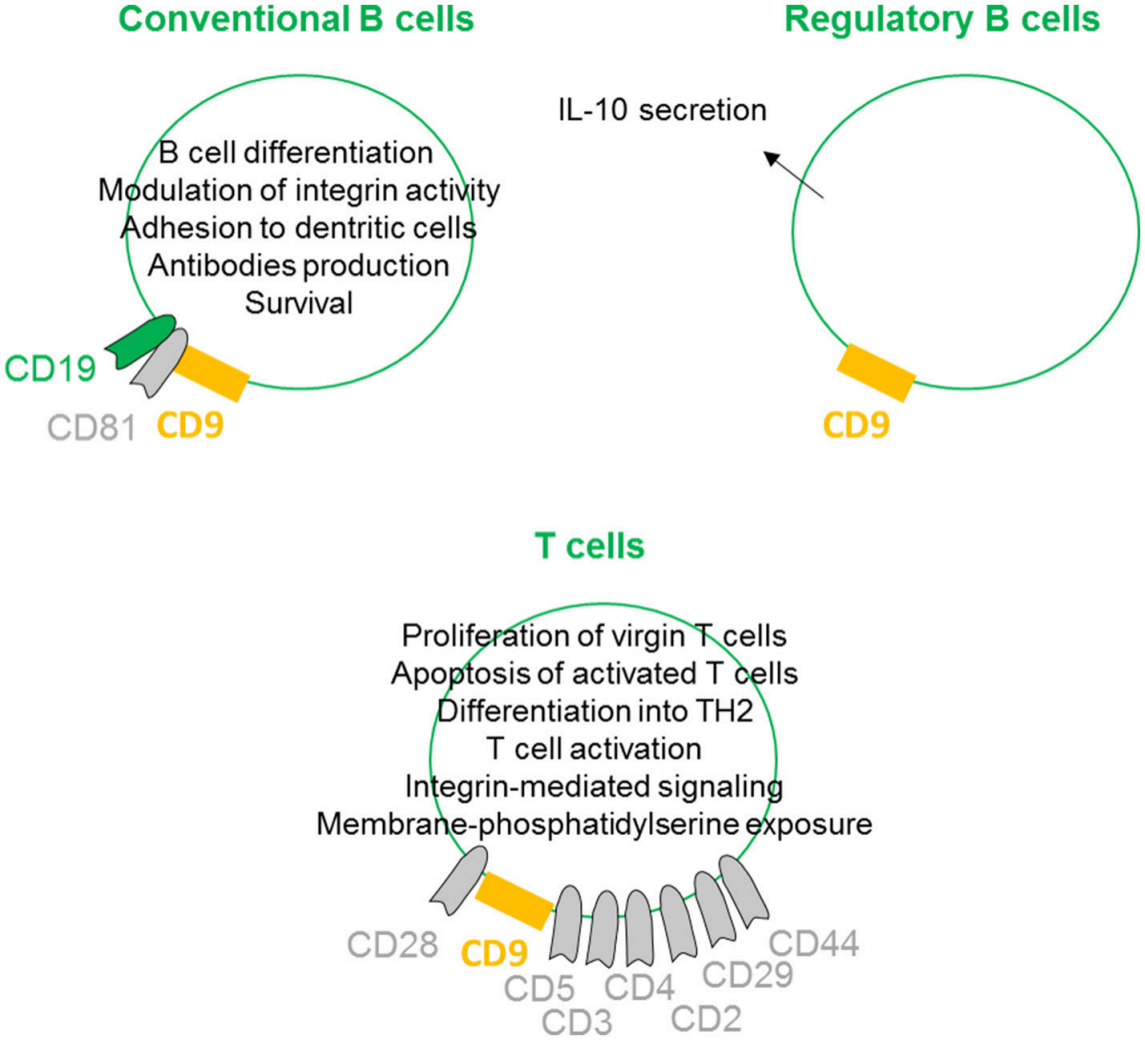
CD9 regulates lymphoid lineages. On conventional B cells, CD9 is physically linked to CD19 through the tetraspan CD81. This association induces B cell differentiation by modulation of integrin activity and allows B cell activities such as antibodies production. CD9 expression also promotes follicular dendritic cell adhesion-inducing survival. CD9 is shown to be involved in the enhancement and maintenance of IL-10 secretion and has been identified as a marker of Breg cells. On T cells, CD9 is associated with CD3, CD4, CD2, CD29, CD44 and more prominently with CD5 and CD28. CD9/CD28 co-ligation induces naïve T cell activation and differentiation into TH2 and apoptosis of activated T cells. CD9 also supports integrin-mediated signaling and can induce membrane-phosphatidylserine exposure.

On conventional B cells, CD9 is expressed during the early stages of differentiation, lost, and re-expressed after B cell activation. CD9 is physically linked to CD19 through the tetraspan CD81 (27) (Figure 4). Indeed, CD9 is co-immunoprecipitated with CD19 from lysates of Brij97 cells line and purified CD10+ early B cell line. This physical interaction is disrupted by diginonin showing that cholesterol participates to the interaction. This association allows the tyrosine phosphorylation of different proteins, suggesting that the CD9/CD19 complex is functional. Because ectopic expression of CD9 stimulates the integrin-dependent motility of the Raji B cell line on fibronectin and laminin substrates, CD9 interferes with B cell differentiation by modulating integrin activity (28). B cells within the germinal center exhibit high heterogeneity, containing B cells at different stages of differentiation. B cells in germinal center tonsils contain two distinct populations expressing or not CD9, with CD9+ B cells being in more advanced stages of plasma cells differentiation (53). Interestingly, CD9 expression enhances the affinity of VLA4 to VCAM-1 on follicular dendritic cells, allowing the strong adhesion of B cells to follicular dendritic cells (29). This tough interaction contributes to better survival of human germinal center B cells. In mouse, CD9 is reported to be a unique marker for B1 and marginal zone B cells and plasma cells (54). In mice deficient in Bruton's tyrosine kinase, the numbers of marginal zone B cells is normal, but these cells and plasma cells express few or no CD9. Thus, CD9 expression is thought to be dependent on signals derived via Bruton's tyrosine kinase, and the high-level expression of CD9 in plasma cells suggest that it may be required for the generation of a functional humoral immune response. However, CD9 is not required for the development of peripheral B cells or for humoral immunity, as B cell development and activation appear normal in CD9-deficient mice (55). There is now ample evidence that some tetraspanins on B-lymphocytes are important in controlling antibody production (30), but because immune cells can express up to 20 different tetraspanin proteins, some of them may likely have a common or redundant function with CD9 on plasma cells.

Regulatory B cells (Breg) are specific subtypes of B cells with immunosuppressive properties. They are involved in the maintenance of homeostasis of the immune system and regulate inflammation in pathological situations (56). Although the presence and role of Bregs are clearly evidenced in different models and pathologies, their full molecular characterization remains elusive, primarily because no specific marker or transcription factor has been identified (57). To date, the most common marker is the ability to secrete IL-10 (58). Several groups are attempting to identify and more precisely define the B cell subtype with suppressive properties among IL10-secreting B cells. We and others have shown that murine IL-10^+^ B cells with suppressive properties are CD9^+^ B cells (59–61). Similarly, Matsushita defined CD9/CD80 co-expression as a novel phenotypic parameter for both Breg subtypes MZ-B10 and B1-B10 cells (62), and CD9 is involved in the enhancement and maintenance of IL-10 secretion in murine and human antigen-presenting cells (23, 31). Finally, we demonstrated that CD9^+^ B cells induce effector T cell growth arrest in sub-G0/G1 and activation of apoptosis via IL-10 (paper under submission). Together, these studies provide evidence that CD9 as a reliable marker for defining both mouse and human Bregs, even if this marker is not generally accepted in the literature yet.

With respect to CD9 expression on T lymphocytes, because CD28-deficient mice can mediate effective T cell-dependent immune responses, Tai and colleagues searched for the existence of other costimulatory systems (39). The authors determined that CD9 is expressed on almost all mature T cells and can deliver a potent CD28-independent costimulatory signal. In contrast to progressive T cell proliferation induced by CD28 co-stimulation, CD9 co-stimulation leads first to proliferation of virgin T cells and second to the induction of apoptosis of once-activated T cells (32). When activated through CD9/CD28 colligation, both CD4^+^ and CD8^+^ naive T cells differentiate into type 2 effector cells, producing IL-2, IL-4, IL-5, IL-13, IL-10, and TNF-α, but produce little or no IFN-γ (33). CD9 is preferentially expressed on the CD4^+^CD45RA^+^ naive T cell subset and is instrumental in both the self-antigen- and recall antigen-induced T cell activation (34). Indeed, anti-CD9 can inhibit both the recombinant beta_2_-glycoprotein I- and the recall antigen tetanus toxoid-specific T cell proliferation, suggesting that the tetraspanin CD9 plays an important role in both T cell activation pathways. CD9 was also found to be associated with CD3, CD4, CD2, CD29, CD44 and more prominently with CD5 (63). To determine the role of CD9 transmembrane or extracellular domains in the association with CD5, CD9 mutant genes lacking each domain were constructed. This study highlights the essential role of particular CD9 transmembrane TM2 and TM3 domains in ligation with CD5. CD9 also congregates at the T-cell side of the immunological synapse between T lymphocytes and antigen-presenting cells supporting integrin-mediated signaling by modulating the relocalization of the α4β1 integrin (26). Finally, CD9 acts selectively on some pathways, leading to membrane-phosphatidylserine exposure, such as stimulation by calcium ionophore, but does not act in response to apoptotic treatments such as ultraviolet light, cycloheximide, or actinomycin D (35).

## CD9 regulates inflammation through endothelial cells

**Figure 5:**
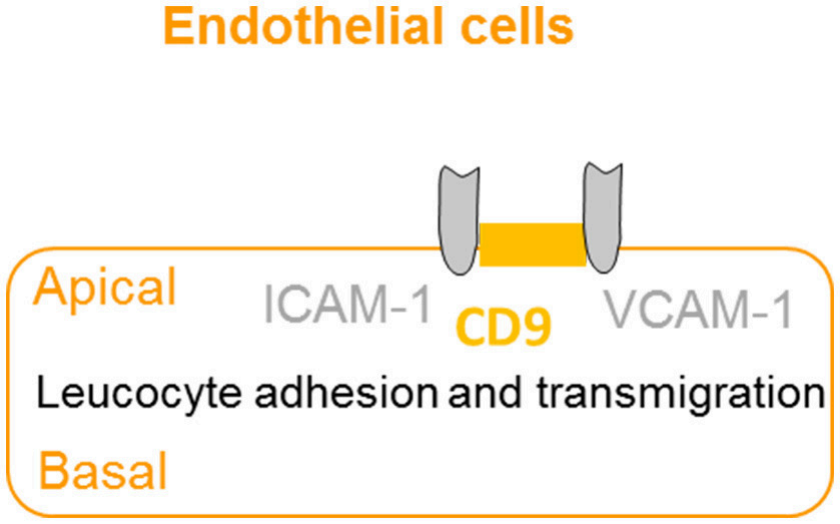
CD9 promotes adhesion and extravasation of leucocyte. CD9 co-localizes with adhesion molecules ICAM-1 and VCAM-1 at the apical endothelial cell surface to promote endothelial–leucocyte adhesion and enhance extravasation and transmigration of leucocytes to the inflammatory site.

The extravasation of leukocytes from the bloodstream to sites of infection involves a complex series of events, which are dependent on interactions between the leucocyte and the apical membrane of the endothelial cells (36). CD9 promotes endothelial–leucocyte adhesion by co-localizing with adhesion molecules ICAM-1 and VCAM-1 at the apical endothelial cell surface, reinforcing the adhesion of the leucocyte to facilitate extravasation (37) (Figure 5). Furthermore, knockdown of CD9 results in reductions of ICAM-1 and VCAM-1 surface expression, thus reducing leucocyte adhesion and transmigration (64). Taken together, CD9 appears as a key regulator of leucocyte recruitment during the inflammatory cascade.

## CD9 is involved in various diseases

### CD9 drives different processes important for viral and bacterial infections

Within TEMs, CD9 modulates various virus-induced processes at the membrane, including membrane fusion, viral budding and viral release, by modifying the structural organization and plasticity of the plasma membrane (65). Human Immunodeficiency Virus type 1 (HIV-1) incorporates specific host cell proteins, including MHC II tetraspanin, CD63, CD9, CD81, and CD82, that are present at the viral exit site in macrophages, dendritic cells and T-cells, and HIV-1 buds through TEMs containing these tetraspanins (66). CD9 negatively regulates HIV-1 Env-induced cell-cell fusion (Env: gp120/gp41). Indeed, CD9 knock-down expression results in an increase of syncytia formation and viral entry; accordingly, overexpression of CD9 renders cells less susceptible to Env-mediated syncytia formation (67). Regarding the mechanisms, CD9 induces hemifusion arrest, blocking HIV-1-induced cell-cell fusion at the transition from hemifusion to pore opening (68). Multimerization of the major HIV-1 structural component Gag at the plasma membrane clusters CD9, restricting its mobility and enhancing virion infectivity. Conversely, CD9 is necessary for several Coronavirus and influenza virus proteolytic priming events, as virus entry can be blocked by CD9 blockade (69). CD9 also promotes adeno-associated virus type 2 infection involvement in the attachment, uptake or processing of this virus (70). Cell–cell contact between plasmacytoid dendritic cells and hepatitis C virus-infected cells is required for the antiviral immune response. The cell surface molecules CD81 and CD9 but no other hepatitis C virus entry receptors are required to recognize hepatitis C virus-infected cells and induce interferon-α (71). Finally, in mice infected with cytopathic foot-and-mouth disease viruses, a rapid and specific thymus-independent neutralizing antibody response is induced to clear the virus. The mechanism highlighted indicates that infected DCs stimulate innate-like CD9+ B lymphocytes from the spleen that are involved in the rapid protection against foot-and-mouth disease virus (72). In conclusion, CD9 has opposing roles in immunity against viruses, on one hand being crucial for anti-infectious immunity, and on the other hand being necessary for some virus priming events.

In the context of bacterial infection, CD9 influences epithelial cell adherence of *Neisseria meningitidis* likely by facilitating specific receptor-adhesin engagement within the TEM. Also, it was shown that CD9 associate through integrins with CD46 (membrane cofactor protein), cofactor that can acts as a receptor for meningococcal allowing its adherence (73). As bacterial adherence is essential for infection, an anti-adhesion therapy based on the use of an anti-CD9 is was tested (74). The expression level of CD9 on each cell type tested was correlated weakly with the efficacy of the inhibitor confirming the crucial role of CD9 in bacterial adherence.

### CD9 has a controversial role in cancer

The role of CD9 in cancer is quite controversial. CD9 expression is sometimes correlated with better survival, and CD9 is sometimes used as a biomarker of invasion and late stages. CD9 is reported to suppress motility and to promote adherence, leading to the suppression of tumor progression. Indeed, CD9 is often downregulated in advanced stages of cancer, and its absence is a sign of poor prognosis in patients with lung, breast, colon, skin, ovary, uterus, stomach, oral cavity, thyroid, prostate, and hematopoietic malignancies (3, 75). However, CD9 is not strictly recognized as a metastasis suppressor gene because in some tumors, inverse activity is observed. For example, in acute lymphoblastic leukemia, CD9 expression indicates a poor outcome (76). The various roles of tetraspanins in tumor progression may be the result of specific tetraspanin abundance in exosomes. A large number of studies have stated a role for exosomes in cancer invasion and metastasis, evasion of apoptosis, drug resistance and escape from immune surveillance (77). Exosomes bear unique protein markers, the main one being CD9 (78). Thus, it is not surprising that CD9 is one of the most useful markers for a range of cancers, with exosomes themselves being reported as a tumor biomarker (79).

### CD9 regulates lung inflammation

CD9 functions as a negative regulator of LPS-induced lung inflammation by activating human monocyte–derived macrophage polarization into a regulatory M2 subset (21). Interestingly, inflammatory agents, such as cigarette smoke extract or trichostatin A, downregulate the expression of CD9 at the surface of macrophages. CD9/CD81 double-knock out mice spontaneously develop a COPD-like phenotype (47). In humans, CD9 expression is lower in blood monocytes from patients with COPD than in those from healthy patients, and CD9 levels are even lower in smokers with COPD than in “healthy smokers” (47). In the field of lung transplantation, we reported a persistently low level of CD9^+^ B cells in patients with chronic lung allograft dysfunction; conversely, patients able to restore a higher level of CD9^+^ B cells posttransplantation were more likely to maintain stable graft function. An increase in CD9^+^ B cells levels between 1 and 24 months after lung transplantation is predictive of long-term survival (patent: EP17305479). Finally, in the context of allergic asthma, there are fewer CD9^+^ B cells in the spleen and lungs of asthmatic mice, and the adoptive transfer of CD9^+^ B cells alone is sufficient to abrogate asthma in an IL-10-dependent manner (59). Interestingly, such IL-10-secreting CD9^+^ B cells are not present in the blood of severe asthmatic patients (paper under submission). In conclusion, CD9 regulates lung inflammation in many diseases, and the absence of CD9 is associated with various dysfunctions, including bronchial hyper-reactivity and chronic lung allograft failure.

**Figure 6:**
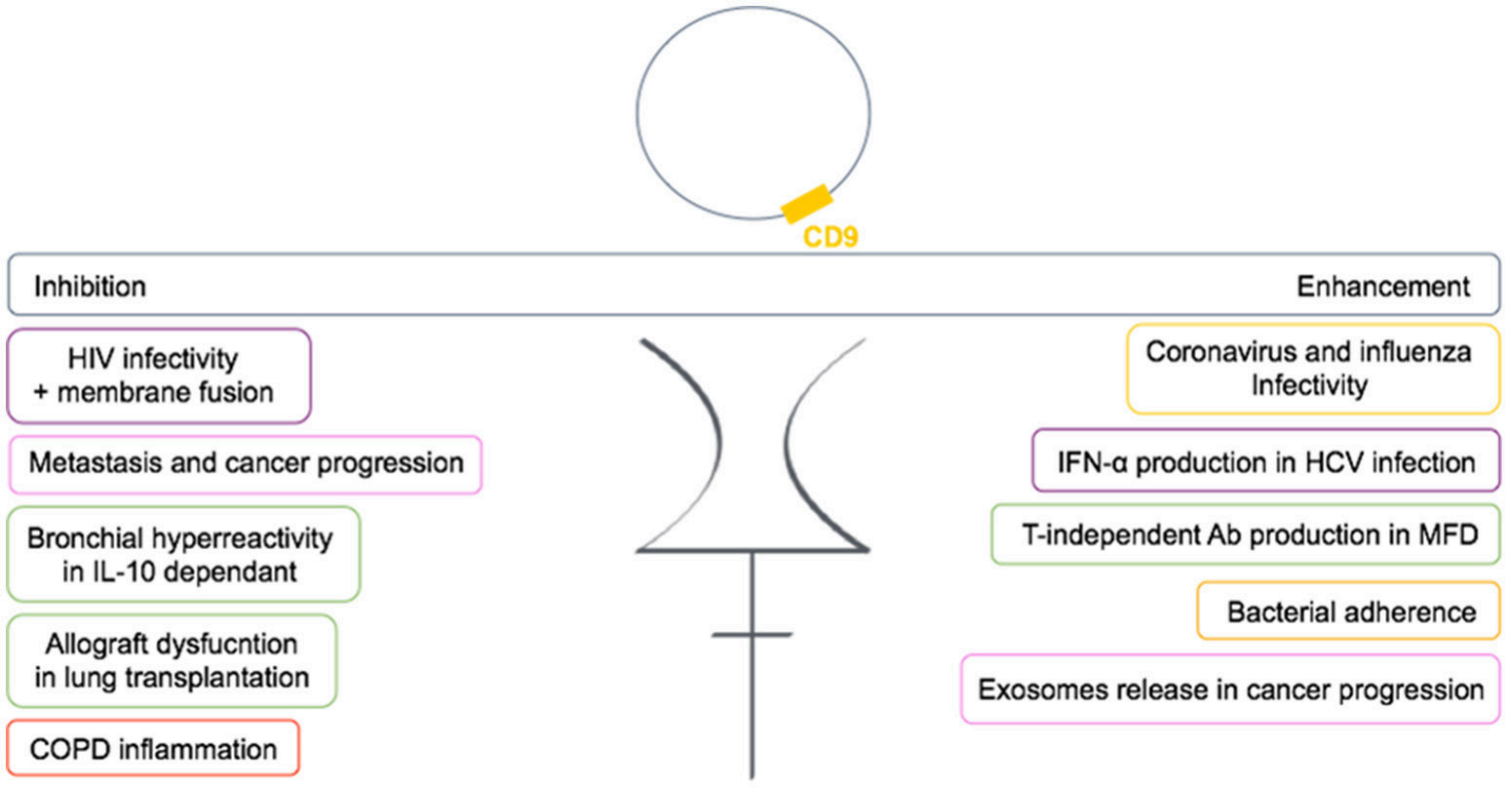
Dual role of CD9 in human diseases. CD9 can either enhance or inhibit pathogenic conditions through TEMs in human diseases. Each color frame refers to implied cells in pathogenic conditions: purple stands for dendritic cells, pink for cancer cells, green for B cells, yellow for epithelial cells, and red for monocytes.

In conclusion, CD9 is functionally very important in many diseases and is involved either in the regulation or in the mediation of the disease (Figure 6). Many studies now suggest CD9 as a biomarker.

## CD9 is a biomarker

CD9 is primarily known today as a specific exosome marker and is used as a specific tool to increase efficiency of exosome extraction methods (78). However, CD9 is also recognized as an anti-inflammatory marker of monocytes and macrophages (49). Indeed, its expression is higher on type II macrophages and lower on pro-inflammatory non-classical monocytes. CD9 is also described as a biomarker of the B lineage. In mice, CD9 is a unique marker for B1 and marginal zone B cells and plasma cells (54), and in humans, CD9 is a marker of plasma cell precursors in germinal centers (53). By transcriptomic analysis, we and others identified CD9 as a marker of murine IL-10-competent Breg cells (59, 60). Finally, we associated IL-10-secreting CD9+ B cells with better allograft outcome in lung transplant patients, and we identified CD9-expressing B cells as a new predictive biomarker of long-term survival (patent: EP17305479). In the field of cancer, CD9 is suggested as a biomarker for metastatic clear cell renal cell carcinoma, not only to distinguish between cancer subtypes but also to predict the metastatic potential of renal cell carcinoma (80, 81). Finally, CD9 is overexpressed in esophageal squamous cell carcinoma tissues and its expression is correlated with tumor stage and lymph node metastasis (82). At the opposite end and in accordance with its controversial role in cancer, CD9 expression in mesothelioma tissue correlates with survival and may be a favorable prognostic marker in patients with mesothelioma (83).

## Targeting CD9 as potential new therapeutic strategy for personalized medicine?

As reviewed here, CD9 is expressed on a wide variety of hematopoietic cells and is diversely involved in the regulation of inflammation. Thus, it seems highly relevant to consider CD9 as a promising therapeutic target.

In the field of cancer, targeting CD9 is widely studied as a potential clinical tool. Interestingly, CD9 is overexpressed on glioblastoma stem cells compared to its expression in healthy brain tissues, opening new therapeutic avenues for CD9 as a target for glioblastoma treatment, especially because xenotransplantation of CD9-silenced glioblastoma stem cells into nude rats promotes prolonged survival (84). Exosomes participate in the communication between neoplastic and normal cells and suppress immune responses and regulate neoplastic growth and metastasis (85). CD9, as constituent of exosomes, could be a potential target for exosome-mediated tumor development. Pro-apoptotic and anti-proliferative properties of the agonist anti-CD9 monoclonal antibody (ABL 6 and PAINS 13) were demonstrated *in vitro* on human malignant cell lines from gastric and colic cancers as well as *in vivo* in SCID mice grafted with those malignant cell lines (86, 87). Taken together, these results suggest an interesting use of agonist anti-CD9 antibodies as therapeutic tools.

Tetraspanins, such as CD9, play a role in anti-viral immunity against HIV. Higher expression of CD9 among other clusters of differentiation is observed on CD4+ and CD8+ circulating lymphocytes from HIV-infected patients with undetectable viral loads and those undergoing highly active antiretroviral therapy. These results are consistent with the negative regulation of HIV infectivity (88). Moreover, CD9 expression is higher in viral niches, including macrophages called VCC (Virus Containing Compartments), arguing for the potential use of CD9 as a therapeutic target and biomarker of those viral niches in combination with CD32a. This enticing strategy could be part of curative strategy for HIV infection (89).

Mast cells belong to innate immunity and are implied in numerous hypersensitivity mechanisms of either combination of allergic, autoimmune, and inflammatory. Many hypersensitivity reactions of either immediate or delayed imply non-IgE mediated mast cell activation/degranulation in which tetraspanins, such as CD9, play a role. Indeed, CD9 dimerize via cross-linking thanks to mAb induces mast cell degranulation (48). CD9 dimerization is suspected to co-localize on the plasma membrane with the high-affinity IgE receptor (FcεRI), leading to mast cell activation and degranulation as observed on a human CD9-transfected basophilic rat leukemia cell line (15). Interestingly, this effect is not found with the Fab' fragment, suggesting an IgE independent activation; CD9 blockade does not prevent Ag-IgE complex mediated degranulation contrary to mAb blocking CD63 and CD81 (90). The main hypothesis is that non-IgE activation/degranulation stresses the role of NTAL TRansmembrane Adaptor Protein (TRAP) (14) or type II IgG receptor (FcγRII), which were recently implicated in IgE-independent anaphylaxis (91). Further studies are needed to evaluate the impact of the non-IgE-mediated mast cell activation/degranulation on drug hypersensitivity or even in chronic urticaria. In asthmatic mice, the adoptive transfer of CD9^+^ B cells normalizes airway inflammation and lung function by inhibiting TH2- and TH17-driven inflammation in an IL-10-dependent manner, which restores a favorable immunological balance in lung tissues (92). Increasing the number of regulatory B cells, including CD9^+^ Breg, is under consideration as an interesting target for the development of new therapies to regulate inflammation in asthma and other allergic diseases to induce allergen tolerance (93–95). Modulation of CD9 expression is even under consideration in auto-immune diseases, such as multiple sclerosis (74), in which blocking CD9 *in vitro* strongly enhances the blood-brain barrier function and reduces the migration of monocytes across brain endothelial cell monolayers.

In the field of respiratory diseases, CD9 appears as a negative regulator of inflammation in COPD and the use of molecules, such as statins, upregulating tetraspanin CD9 in macrophages and allowing decreases of inflammation are under consideration (96) for therapeutic intervention. Finally, CD9^+^ B cells secreting IL-10 are associated with better lung allograft outcomes after lung transplantation through creating a favorable environment for the graft by reducing the inflammation leading to chronic lung dysfunction. Some immunosuppressive treatments, such as anti-calcineurin, mTOR inhibitors, and tacrolimus, used in lung transplanted patients reduce some Bregs subsets (97, 98). Conversely, Belatacept favors the survival of B cells with regulatory properties. Thus, the use of immunosuppressive treatments in lung-transplanted patients that have no effect on the Breg CD9+ compartment may be essential to maintain the level of circulating Breg CD9+ and to reduce the risk of graft dysfunction.

Finally, anti-CD9 is also tested for its anti-adhesive properties to inhibit bacterial adhesion to keratinocytes and has been shown to be effective in a tissue-engineered model of human skin infected with *Staphylococcus aureus* suggesting that CD9 inhibitors may be a valuable addition to current treatments of skin infection and underscore the potential of targeting CD9 in bacterial infectious diseases (99).

## Concluding remarks

In conclusion, CD9 is expressed on a wide variety of hematopoietic cells, allowing the modulation of a large range of pathways to regulate inflammation. Modeling novel immunotherapies based on tetraspanin modulation is a two-faceted strategy. Indeed, the plasma membrane compartmentalization properties in TEMs are of primary interest because the location allows modulation of immune receptor activity. However, the ubiquitous tissue distribution of tetraspanins and their potential functional redundancy are the main obstacles in therapeutic strategy development focusing on TEMs. Furthermore, depending on the molecule-associated partners or cell type expression, CD9 can have opposite effects. Nevertheless, depending on the context, the environment and the pathology, modulating CD9 expression or blocking its effects seem to be a new promising therapeutic strategy that deserves further investigation.

## Author contributions

All authors listed have made a substantial, direct and intellectual contribution to the work, and approved it for publication.

### Conflict of interest statement

The authors declare that the research was conducted in the absence of any commercial or financial relationships that could be construed as a potential conflict of interest.

## References

[1] Berditchevski F Odintsova E. Tetraspanins as regulators of protein trafficking. Traffic (2007) 8:89–96. 10.1111/j.1600-0854.2006.00515.x 17181773

[2] Termini CM Gillette JM. Tetraspanins function as regulators of cellular signaling. Front Cell Dev Biol. (2017) 5:34. 10.3389/fcell.2017.00034 28428953 PMC5382171

[3] Boucheix C Duc GH Jasmin C Rubinstein E. Tetraspanins and malignancy. Expert Rev Mol Med. (2001) 2001:1–17. 10.1017/S1462399401002381 14987371

[4] Levy S Shoham T. The tetraspanin web modulates immune-signalling complexes. Nat Rev Immunol. (2005) 5:136–148. 10.1038/nri1548 15688041

[5] Seipold L Saftig P. The emerging role of tetraspanins in the proteolytic processing of the amyloid precursor protein. Front Mol Neurosci. (2016) 9:149. 10.3389/fnmol.2016.00149 28066176 PMC5174118

[6] Hadjiargyrou M Patterson PH. An anti-CD9 monoclonal antibody promotes adhesion and induces proliferation of Schwann cells *in vitro*. J Neurosci. (1995) 15:574–83. 7823165 10.1523/JNEUROSCI.15-01-00574.1995 PMC6578267

[7] Powner D Kopp PM Monkley SJ Critchley DR Berditchevski F. Tetraspanin CD9 in cell migration. Biochem Soc Trans. (2011) 39:563–7. 10.1042/BST0390563 21428940

[8] Le Naour F Rubinstein E Jasmin C Prenant M Boucheix C Severely reduced female fertility in CD9-deficient mice. Science (2000) 287:319–21. 10.1126/science.287.5451.319 10634790

[9] Clay D Rubinstein E Mishal Z Anjo A Prenant M Jasmin C. CD9 and megakaryocyte differentiation. Blood (2001) 97:1982–9. 10.1182/blood.V97.7.1982 11264162

[10] Oritani K Wu X Medina K Hudson J Miyake K Gimble JM. Antibody ligation of CD9 modifies production of myeloid cells in long-term cultures. Blood (1996) 87:2252–61. 8630385

[11] Aoyama K Oritani K Yokota T Ishikawa J Nishiura T Miyake K. Stromal cell CD9 regulates differentiation of hematopoietic stem/progenitor cells. Blood (1999) 93:2586–94. 10194438

[12] Qi JC Wang J Mandadi S Tanaka K Roufogalis BD Madigan MC. Human and mouse mast cells use the tetraspanin CD9 as an alternate interleukin-16 receptor. Blood (2006) 107:135–142. 10.1182/blood-2005-03-1312 16144798 PMC1895361

[13] Redegeld FA Yu Y Kumari S Charles N Blank U. Non-IgE mediated mast cell activation. Immunol Rev. (2018) 282:87–113. 10.1111/imr.12629 29431205

[14] Hálová I Dráberová L Bambousková M Machyna M Stegurová L Smrz D. Cross-talk between tetraspanin CD9 and transmembrane adaptor protein non-T cell activation linker (NTAL) in mast cell activation and chemotaxis. J Biol Chem. (2013) 288:9801–14. 10.1074/jbc.M112.449231 23443658 PMC3617281

[15] Higginbottom A Wilkinson I McCullough B Lanza F Azorsa DO Partridge LJ. Antibody cross-linking of human CD9 and the high-affinity immunoglobulin E receptor stimulates secretion from transfected rat basophilic leukaemia cells. Immunology (2000) 99:546–52. 10.1046/j.1365-2567.2000.00992.x 10792502 PMC2327194

[16] Fernvik E Halldén G Hed J Lundahl J. Intracellular and surface distribution of CD9 in human eosinophils. APMIS (1995) 103:699–706. 8534428 10.1111/j.1699-0463.1995.tb01426.x

[17] Kim JT Gleich GJ Kita H. Roles of CD9 molecules in survival and activation of human eosinophils. J Immunol. (1997) 159:926–933. 9218613

[18] Akuthota P Melo RCN Spencer LA Weller PF. MHC Class II and CD9 in human eosinophils localize to detergent-resistant membrane microdomains. Am J Respir Cell Mol Biol. (2012) 46:188–95. 10.1165/rcmb.2010-0335OC 21885678 PMC3297164

[19] Bandeira-Melo C Perez SAC Melo RCN Ghiran I Weller PF. EliCell assay for the detection of released cytokines from eosinophils. J Immunol Methods (2003) 276:227–237. 10.1016/S0022-1759(03)00076-0 12738376

[20] Qi R Ozaki Y Kuroda K Asazuma N Yatomi Y Satoh K. Differential activation of human platelets induced by Fc gamma receptor II cross-linking and by anti-CD9 monoclonal antibody. J Immunol. (1996) 157:5638–45. 8955216

[21] Suzuki M Tachibana I Takeda Y He P Minami S Iwasaki T. Tetraspanin CD9 negatively regulates lipopolysaccharide-induced macrophage activation and lung inflammation. J Immunol. (2009) 182:6485–93. 10.4049/jimmunol.0802797 19414803

[22] Kaji K Takeshita S Miyake K Takai T Kudo A. Functional association of CD9 with the Fc gamma receptors in macrophages. J Immunol. (2001) 166:3256–65. 10.1128/microbiolspec.MCHD-0045-2016 11207280

[23] Ha CT Waterhouse R Wessells J Wu JA Dveksler GS. Binding of pregnancy-specific glycoprotein 17 to CD9 on macrophages induces secretion of IL-10, IL-6, PGE2, and TGF-beta1. J Leukoc Biol. (2005) 77:948–57. 10.1189/jlb.0804453 15772125

[24] Huang W Febbraio M Silverstein RL. CD9 tetraspanin interacts with CD36 on the surface of macrophages: a possible regulatory influence on uptake of oxidized low density lipoprotein. PLoS ONE (2011) 6:e29092. 10.1371/journal.pone.0029092 22216174 PMC3244426

[25] Zilber M-T Setterblad N Vasselon T Doliger C Charron D Mooney N. MHC class II/CD38/CD9: a lipid-raft-dependent signaling complex in human monocytes. Blood (2005) 106:3074–81. 10.1182/blood-2004-10-4094 15941914

[26] Rocha-Perugini V González-Granado JM Tejera E López-Martín S Yañez-Mó M Sánchez-Madrid F. Tetraspanins CD9 and CD151 at the immune synapse support T-cell integrin signaling. Eur J Immunol. (2014) 44:1967–75. 10.1002/eji.201344235 24723389 PMC4630866

[27] Horváth G Serru V Clay D Billard M Boucheix C Rubinstein E. CD19 is linked to the integrin-associated tetraspans CD9, CD81, and CD82. J Biol Chem. (1998) 273:30537–43. 9804823 10.1074/jbc.273.46.30537

[28] Shaw AR Domanska A Mak A Gilchrist A Dobler K Visser L. Ectopic expression of human and feline CD9 in a human B cell line confers beta 1 integrin-dependent motility on fibronectin and laminin substrates and enhanced tyrosine phosphorylation. J Biol Chem. (1995) 270:24092–9. 7592610 10.1074/jbc.270.41.24092

[29] Yoon S-O Lee IY Zhang X Zapata MC Choi YS. CD9 may contribute to the survival of human germinal center B cells by facilitating the interaction with follicular dendritic cells. FEBS Open Biol. (2014) 4:370–6. 10.1016/j.fob.2014.04.001 24918051 PMC4050195

[30] van Spriel AB. Tetraspanins in the humoral immune response. Biochem Soc Trans. (2011) 39:512–7. 10.1042/BST0390512 21428930

[31] Kabuto M Fujimoto N Takahashi T Tanaka T. Decreased level of interleukin-10-producing B cells in patients with pemphigus but not in patients with pemphigoid. Br J Dermatol. (2017) 176:1204–12. 10.1111/bjd.15113 27716906

[32] Tai XG Toyooka K Yashiro Y Abe R Park CS Hamaoka T. CD9-mediated costimulation of TCR-triggered naive T cells leads to activation followed by apoptosis. J Immunol. (1997) 159:3799–807. 9378967

[33] Serra A Nuti S Tavarini S Sammicheli C Rosa D Saletti G. Coligation of the hepatitis C virus receptor CD81 with CD28 primes naive T lymphocytes to acquire type 2 effector function. J Immunol. (2008) 181:174–85. 10.4049/jimmunol.181.1.174 18566382

[34] Kobayashi H Hosono O Iwata S Kawasaki H Kuwana M Tanaka H. The tetraspanin CD9 is preferentially expressed on the human CD4(+)CD45RA+ naive T cell population and is involved in T cell activation. Clin Exp Immunol. (2004) 137:101–8. 10.1111/j.1365-2249.2004.02494.x 15196249 PMC1809091

[35] Li W Tait JF. Regulatory effect of CD9 on calcium-stimulated phosphatidylserine exposure in Jurkat T lymphocytes. Arch Biochem Biophys. (1998) 351:89–95. 10.1006/abbi.1997.0535 9500845

[36] Nourshargh S Hordijk PL Sixt M. Breaching multiple barriers: leukocyte motility through venular walls and the interstitium. Nat Rev Mol Cell Biol. (2010) 11:366–78. 10.1038/nrm2889 20414258

[37] Bailey RL Herbert JM Khan K Heath VL Bicknell R Tomlinson MG. The emerging role of tetraspanin microdomains on endothelial cells. Biochem Soc Trans. (2011) 39:1667–73. 10.1042/BST20110745 22103505

[38] Kersey JH LeBien TW Abramson CS Newman R Sutherland R Greaves M. P-24: a human leukemia-associated and lymphohemopoietic progenitor cell surface structure identified with monoclonal antibody. J Exp Med. (1981) 153:726–731. 6788880 10.1084/jem.153.3.726 PMC2186112

[39] Tai XG Yashiro Y Abe R Toyooka K Wood CR Morris J. A role for CD9 molecules in T cell activation. J Exp Med. (1996) 184:753–758. 8760830 10.1084/jem.184.2.753 PMC2192734

[40] Miyake M Koyama M Seno M Ikeyama S. Identification of the motility-related protein (MRP-1), recognized by monoclonal antibody M31-15, which inhibits cell motility. J Exp Med. (1991) 174:1347–54. 1720807 10.1084/jem.174.6.1347 PMC2119050

[41] Waterhouse R Ha C Dveksler GS. Murine CD9 is the receptor for pregnancy-specific glycoprotein 17. J Exp Med. (2002) 195:277–282. 10.1084/jem.20011741 11805154 PMC2193606

[42] Chen MS Tung KS Coonrod SA Takahashi Y Bigler D Chang A. Role of the integrin-associated protein CD9 in binding between sperm ADAM 2 and the egg integrin alpha6beta1: implications for murine fertilization. Proc Natl Acad Sci USA. (1999) 96:11830–5. 10518536 10.1073/pnas.96.21.11830 PMC18372

[43] Inoue N Hamada D Kamikubo H Hirata K Kataoka M Yamamoto M. Molecular dissection of IZUMO1, a sperm protein essential for sperm-egg fusion. Development (2013) 140:3221–9. 10.1242/dev.094854 23824580

[44] Jégou A Ziyyat A Barraud-Lange V Perez E Wolf JP Pincet F. CD9 tetraspanin generates fusion competent sites on the egg membrane for mammalian fertilization. Proc Natl Acad Sci USA. (2011) 108:10946–51. 10.1073/pnas.1017400108 21690351 PMC3131345

[45] Rappa G Green TM Karbanová J Corbeil D Lorico A. Tetraspanin CD9 determines invasiveness and tumorigenicity of human breast cancer cells. Oncotarget (2015) 6:7970–91. 10.18632/oncotarget.3419 25762645 PMC4480729

[46] Sumiyoshi N Ishitobi H Miyaki S Miyado K Adachi N Ochi M. The role of tetraspanin CD9 in osteoarthritis using three different mouse models. Biomed Res. (2016) 37:283–291. 10.2220/biomedres.37.283 27784871

[47] Takeda Y Suzuki M Jin Y Tachibana I. Preventive Role of Tetraspanin CD9 in Systemic Inflammation of Chronic Obstructive Pulmonary Disease. Am J Respir Cell Mol Biol. (2015) 53:751–60. 10.1165/rcmb.2015-0122TR 26378766

[48] Halova I Draber P. Tetraspanins and transmembrane adaptor proteins as plasma membrane organizers-mast cell case. Front Cell Dev Biol. (2016) 4:43. 10.3389/fcell.2016.00043 27243007 PMC4861716

[49] Tippett E Cameron PU Marsh M Crowe SM. Characterization of tetraspanins CD9, CD53, CD63, and CD81 in monocytes and macrophages in HIV-1 infection. J Leukoc Biol. (2013) 93:913–20. 10.1189/jlb.0812391 23570947

[50] Wang X-Q Evans GF Alfaro ML Zuckerman SH. Down-regulation of macrophage CD9 expression by interferon-gamma. Biochem Biophys Res Commun. (2002) 290:891–7. 10.1006/bbrc.2001.6293 11798156

[51] Peng WM Yu CF Kolanus W Mazzocca A Bieber T Kraft S. Tetraspanins CD9 and CD81 are molecular partners of trimeric FcεRI on human antigen-presenting cells. Allergy (2011) 66:605–11. 10.1111/j.1398-9995.2010.02524.x 21241315

[52] Morelli AE Larregina AT Shufesky WJ Sullivan MLG Stolz DB Papworth GD. Endocytosis, intracellular sorting, and processing of exosomes by dendritic cells. Blood (2004) 104:3257–66. 10.1182/blood-2004-03-0824 15284116

[53] Yoon S-O Zhang X Lee IY Spencer N Vo P Choi YS. CD9 is a novel marker for plasma cell precursors in human germinal centers. Biochem Biophys Res Commun. (2013) 431:41–6. 10.1016/j.bbrc.2012.12.102 23291167 PMC3563937

[54] Won W-J Kearney JF. CD9 is a unique marker for marginal zone B cells, B1 cells, and plasma cells in mice. J Immunol. (2002) 168:5605–5611. 10.4049/jimmunol.168.11.5605 12023357

[55] Cariappa A Shoham T Liu H Levy S Boucheix C Pillai S The CD9 tetraspanin is not required for the development of peripheral B cells or for humoral immunity. J Immunol. (2005) 175:2925–30. 10.4049/jimmunol.175.5.2925 16116178

[56] Wolf SD Dittel BN Hardardottir F Janeway CA. Experimental autoimmune encephalomyelitis induction in genetically B cell-deficient mice. J Exp Med. (1996) 184:2271–8. 8976182 10.1084/jem.184.6.2271 PMC2196394

[57] Mauri C Menon M. The expanding family of regulatory B cells. Int Immunol. (2015) 27:479–86. 10.1093/intimm/dxv038 26071023 PMC4587489

[58] Fillatreau S Sweenie CH McGeachy MJ Gray D Anderton SM. B cells regulate autoimmunity by provision of IL-10. Nat Immunol. (2002) 3:944–950. 10.1038/ni833 12244307

[59] Braza F Chesne J Durand M Dirou S Brosseau C Mahay G. A regulatory CD9(+) B-cell subset inhibits HDM-induced allergic airway inflammation. Allergy (2015) 70:1421–31. 10.1111/all.12697 26194936

[60] Sun J Wang J Pefanis E Chao J Rothschild G Tachibana I. Transcriptomics Identify CD9 as a marker of murine IL-10-competent regulatory B cells. Cell Rep. (2015) 13:1110–7. 10.1016/j.celrep.2015.09.070 26527007 PMC4644501

[61] Said SS Barut GT Mansur N Korkmaz A Sayi-Yazgan A. Bacterially activated B-cells drive T cell differentiation towards Tr1 through PD-1/PD-L1 expression. Mol Immunol. (2018) 96:48–60. 10.1016/j.molimm.2018.02.010 29494848

[62] Matsushita T Le Huu D Kobayashi T Hamaguchi Y Hasegawa M Naka K. A novel splenic B1 regulatory cell subset suppresses allergic disease through phosphatidylinositol 3-kinase-Akt pathway activation. J Allergy Clin Immunol. (2016) 138:1170–82.e9. 10.1016/j.jaci.2015.12.1319 26948079

[63] Toyo-oka K Yashiro-Ohtani Y Park CS Tai XG Miyake K Hamaoka T. Association of a tetraspanin CD9 with CD5 on the T cell surface: role of particular transmembrane domains in the association. Int Immunol. (1999) 11:2043–52. 10590270 10.1093/intimm/11.12.2043

[64] Barreiro O Yáñez-Mó M Sala-Valdés M Gutiérrez-López MD Ovalle S Higginbottom A. Endothelial tetraspanin microdomains regulate leukocyte firm adhesion during extravasation. Blood (2005) 105:2852–61. 10.1182/blood-2004-09-3606 15591117

[65] Singethan K Müller N Schubert S Lüttge D Krementsov DN Khurana SR. CD9 clustering and formation of microvilli zippers between contacting cells regulates virus-induced cell fusion. Traffic (2008) 9:924–35. 10.1111/j.1600-0854.2008.00737.x 18363777 PMC2992846

[66] Thali M. Tetraspanin functions during HIV-1 and influenza virus replication. Biochem Soc Trans. (2011) 39:529–31. 10.1042/BST0390529 21428933 PMC4067976

[67] Gordón-Alonso M Yañez-Mó M Barreiro O Alvarez S Muñoz-Fernández MA Valenzuela-Fernández A. Tetraspanins CD9 and CD81 modulate HIV-1-induced membrane fusion. J Immunol. (2006) 177:5129–5137. 10.4049/jimmunol.177.8.5129 17015697

[68] Symeonides M Lambelé M Roy NH Thali M. Evidence showing that tetraspanins inhibit HIV-1-induced cell-cell fusion at a post-hemifusion stage. Viruses (2014) 6:1078–90. 10.3390/v6031078 24608085 PMC3970140

[69] Earnest JT Hantak MP Park J-E Gallagher T. Coronavirus and influenza virus proteolytic priming takes place in tetraspanin-enriched membrane microdomains. J Virol. (2015) 89:6093–104. 10.1128/JVI.00543-15 25833045 PMC4442435

[70] Kurzeder C Koppold B Sauer G Pabst S Kreienberg R Deissler H. CD9 promotes adeno-associated virus type 2 infection of mammary carcinoma cells with low cell surface expression of heparan sulphate proteoglycans. Int J Mol Med. (2007) 19:325–333. 10.3892/ijmm.19.2.325 17203208

[71] Zhang S Kodys K Babcock GJ Szabo G. CD81/CD9 tetraspanins aid plasmacytoid dendritic cells in recognition of hepatitis C virus-infected cells and induction of interferon-alpha. Hepatology (2013) 58:940–9. 10.1002/hep.25827 22577054 PMC4511847

[72] Ostrowski M Vermeulen M Zabal O Zamorano PI Sadir AM Geffner JR. The early protective thymus-independent antibody response to foot-and-mouth disease virus is mediated by splenic CD9+ B lymphocytes. J Virol. (2007) 81:9357–67. 10.1128/JVI.00677-07 17567692 PMC1951431

[73] Green LR Monk PN Partridge LJ Morris P Gorringe AR Read RC. Cooperative role for tetraspanins in adhesin-mediated attachment of bacterial species to human epithelial cells. Infect Immun. (2011) 79:2241–9. 10.1128/IAI.01354-10 21464080 PMC3125835

[74] Schenk GJ Dijkstra S van het Hof AJ van der Pol SMA Drexhage JAR van der Valk P. Roles for HB-EGF and CD9 in multiple sclerosis. Glia (2013) 61:1890–905. 10.1002/glia.22565 24038577

[75] Wang H-X Li Q Sharma C Knoblich K Hemler ME. Tetraspanin protein contributions to cancer. Biochem Soc Trans. (2011) 39:547–52. 10.1042/BST0390547 21428937

[76] Liang P Miao M Liu Z Wang H Jiang W Ma S. CD9 expression indicates a poor outcome in acute lymphoblastic leukemia. Cancer Biomark (2018) 21:781–6. 10.3233/CBM-170422 29286918 PMC13078320

[77] Zöller M. Tetraspanins: push and pull in suppressing and promoting metastasis. Nat Rev Cancer (2009) 9:40–55. 10.1038/nrc2543 19078974

[78] Caradec J Kharmate G Hosseini-Beheshti E Adomat H Gleave M Guns E. Reproducibility and efficiency of serum-derived exosome extraction methods. Clin Biochem. (2014) 47:1286–92. 10.1016/j.clinbiochem.2014.06.011 24956264

[79] Soung YH Ford S Zhang V Chung J. Exosomes in Cancer Diagnostics. Cancers (2017) 9:8. 10.3390/cancers9010008 28085080 PMC5295779

[80] Kwon HJ Min SY Park MJ Lee C Park JH Chae JY. Expression of CD9 and CD82 in clear cell renal cell carcinoma and its clinical significance. Pathol Res Pract. (2014) 210:285–90. 10.1016/j.prp.2014.01.004 24553302

[81] Garner JM Herr MJ Hodges KB Jennings LK. The utility of tetraspanin CD9 as a biomarker for metastatic clear cell renal cell carcinoma. Biochem Biophys Res Commun. (2016) 471:21–25. 10.1016/j.bbrc.2016.02.008 26855131

[82] Huan J Gao Y Xu J Sheng W Zhu W Zhang S. Overexpression of CD9 correlates with tumor stage and lymph node metastasis in esophageal squamous cell carcinoma. Int J Clin Exp Pathol. (2015) 8:3054–61. 26045817 PMC4440126

[83] Amatya VJ Takeshima Y Aoe K Fujimoto N Okamoto T Yamada T. CD9 expression as a favorable prognostic marker for patients with malignant mesothelioma. Oncol Rep. (2013) 29:21–8. 10.3892/or.2012.2116 23128478 PMC3583601

[84] Podergajs N Motaln H Rajčević U Verbovšek U Koršič M Obad N. Transmembrane protein CD9 is glioblastoma biomarker, relevant for maintenance of glioblastoma stem cells. Oncotarget (2016) 7:593–609. 10.18632/oncotarget.5477 26573230 PMC4808020

[85] Kumar D Gupta D Shankar S Srivastava RK. Biomolecular characterization of exosomes released from cancer stem cells: possible implications for biomarker and treatment of cancer. Oncotarget (2015) 6:3280–91. 10.18632/oncotarget.2462 25682864 PMC4413653

[86] Murayama Y Oritani K Tsutsui S. Novel CD9-targeted therapies in gastric cancer. World J Gastroenterol. (2015) 21:3206–13. 10.3748/wjg.v21.i11.3206 25805926 PMC4363749

[87] Nakamoto T Murayama Y Oritani K Boucheix C Rubinstein E Nishida M. A novel therapeutic strategy with anti-CD9 antibody in gastric cancers. J Gastroenterol. (2009) 44:889–96. 10.1007/s00535-009-0081-3 19468669

[88] Wu JQ Dyer WB Chrisp J Belov L Wang B Saksena NK. Longitudinal microarray analysis of cell surface antigens on peripheral blood mononuclear cells from HIV+ individuals on highly active antiretroviral therapy. Retrovirology (2008) 5:24. 10.1186/1742-4690-5-24 18315888 PMC2276515

[89] Descours B Petitjean G López-Zaragoza J-L Bruel T Raffel R Psomas C. CD32a is a marker of a CD4 T-cell HIV reservoir harbouring replication-competent proviruses. Nature (2017) 543:564–7. 10.1038/nature21710 28297712

[90] Kraft S Fleming T Billingsley JM Lin S-Y Jouvin M-H Storz P. Anti-CD63 antibodies suppress IgE-dependent allergic reactions *in vitro* and *in vivo*. J Exp Med. (2005) 201:385–96. 10.1084/jem.20042085 15684326 PMC2213034

[91] Francis A Bosio E Stone SF Fatovich DM Arendts G Nagree Y. Neutrophil activation during acute human anaphylaxis: analysis of MPO and sCD62L. Clin Exp Allergy (2017) 47:361–70. 10.1111/cea.12868 27906487

[92] Braza F Chesne J Castagnet S Magnan A Brouard S. Regulatory functions of B cells in allergic diseases. Allergy (2014) 69:1454–63. 10.1111/all.12490 25060230

[93] Dong J Wong CK Cai Z Jiao D Chu M Lam CWK. Amelioration of allergic airway inflammation in mice by regulatory IL-35 through dampening inflammatory dendritic cells. Allergy (2015) 70:921–32. 10.1111/all.12631 25869299

[94] Blair PA Chavez-Rueda KA Evans JG Shlomchik MJ Eddaoudi A Isenberg DA. Selective targeting of B cells with agonistic anti-CD40 is an efficacious strategy for the generation of induced regulatory T2-like B cells and for the suppression of lupus in MRL/lpr mice. J Immunol. (2009) 182:3492–502. 10.4049/jimmunol.0803052 19265127 PMC4082659

[95] Korczak-Kowalska G Stelmaszczyk-Emmel A Bocian K Kiernozek E Drela N Domagała-Kulawik J. Expanding diversity and common goal of regulatory T and B cells. II: in allergy, malignancy, and transplantation. Arch Immunol Ther Exp. (2017) 65:523–35. 10.1007/s00005-017-0471-9 28470464 PMC5688211

[96] Jin Y Tachibana I Takeda Y He P Kang S Suzuki M. Statins decrease lung inflammation in mice by upregulating tetraspanin CD9 in macrophages. PLoS ONE (2013) 8:e73706. 10.1371/journal.pone.0073706 24040034 PMC3767596

[97] Tebbe B Wilde B Ye Z Wang J Wang X Jian F. Renal transplant recipients treated with calcineurin-inhibitors lack circulating immature transitional CD19+CD24hiCD38hi regulatory b-lymphocytes. PLoS ONE (2016) 11:e0153170. 10.1371/journal.pone.0153170 27045291 PMC4821620

[98] Chung BH Kim KW Yu JH Kim B-M Choi BS Park CW. Decrease of immature B cell and interleukin-10 during early-post-transplant period in renal transplant recipients under tacrolimus based immunosuppression. Transpl Immunol. (2014) 30:159–67. 10.1016/j.trim.2014.03.003 24709525

[99] Ventress JK Partridge LJ Read RC Cozens D MacNeil S Monk PN. Peptides from tetraspanin CD9 are potent inhibitors of *Staphylococcus aureus* adherence to keratinocytes. PLoS ONE (2016) 11:e0160387. 10.1371/journal.pone.0160387 27467693 PMC4965146

